# Evaluation of Osteoconductive and Osteogenic Potential of a Dentin-Based Bone Substitute Using a Calvarial Defect Model

**DOI:** 10.1155/2012/396316

**Published:** 2012-03-15

**Authors:** Ibrahim Hussain, Keyvan Moharamzadeh, Ian M. Brook, Patrício José de Oliveira Neto, Luiz A. Salata

**Affiliations:** ^1^School of Clinical Dentistry, The University of Sheffield, Claremont Crescent, Sheffield S10 2TA, UK; ^2^Department of Oral and Maxillofacial Surgery and Periodontics, Faculty of Dentistry of Ribeirao Preto, University of São Paulo, Avenida do Cafe s/n—Campus USP, 14040-904 Ribeirão Preto, SP, Brazil

## Abstract

The aim of this study was to assess the osteoconductive and osteogenic properties of processed bovine dentin using a robust rabbit calvarial defect model. In total, 16 New Zealand White rabbits were operated to create three circular defects in the calvaria. One defect was left unfilled, one filled with collected autogenous bone, and the third defect was filled with the dentin-based bone substitute. Following surgery and after a healing period of either 1 or 6 weeks, a CT scan was obtained. Following sacrificing, the tissues were processed for histological examination. The CT data showed the density in the area grafted with the dentin-based material was higher than the surrounding bone and the areas grafted with autologous bone after 1 week and 6 weeks of healing. The area left unfilled remained an empty defect after 1 week and 6 weeks. Histological examination of the defects filled with the dentin product after 6 weeks showed soft tissue encapsulation around the dentin particles. It can be concluded that the rabbit calvarial model used in this study is a robust model for the assessment of bone materials. Bovine dentin is a biostable material; however, it may not be suitable for repairing large 4-wall defects.

## 1. Introduction

Allogenic dentin has been studied for its potential use as bone substitute [1–4]. Previously, a new method for processing bovine dentin was reported that resulted in a sterile bioactive material for repair and regeneration of bone [5]. This included processing the extracted bovine dentin mechanically and chemically with inorganic and organic solvents. 

Initial biocompatibility tests involved *in vitro* testing on human gingival fibroblasts using the Alamar Blue assay and *in vivo* evaluation by implantation of the processed dentin into rat femur. The dentin product showed excellent biocompatibility in the rat femur model and stimulated the formation of new bone and was completely integrated with the surrounding bone. However, the rat femur model used in the previous study was a small 5-wall defect healing model. Healing in rat defects is known to be well underway by 9 weeks postoperatively. If the defect is 2 mm, then the defect will heal by bony union. If the defect is above 5 mm, then fibrous nonunion healing will occur [6]. The rat femur implantation test has high osteogenic potential due to its 5 bony walls that are mainly cancellous bone with surrounding marrow.

To obtain a rigorous assessment of the osteoconductive and osteogenic potential of the new dentin-based material, a robust rabbit calvarial defect model with 4-wall defects is preferred. The rabbit calvarial model is different from rat femur model as the defect in rabbit calvarium is similar to those created in the maxillofacial region in human, since morphologically and embryologically calvarium develops from a membrane precursor resembling the membranous bones of the face [7]. The defect has only 4 bony walls, and the surrounding bone is mainly cortical bone with a poorer blood supply and less osteogenic cells being presented to the defect. Thus, it is regarded as a more robust model than the rat femur model.

The aim of this study was to assess the biocompatibility of the previously developed bovine dentin product using a robust rabbit calvarial defect model.

## 2. Materials and Methods

### 2.1. Ethical Approval

This study was approved by the University of Sao Paulo's Animal Research Ethics Committee. The animal experiments were carried out in the Department of Oral Maxillofacial Surgery, University of Sao Paulo in Brazil.

### 2.2. Bovine Dentin Processing

Extracted bovine dentin was processed mechanically and chemically with inorganic and organic solvents to produce a sterile powder with mixed particle size. Methods are described in detail in our previous report [5].

### 2.3. Animals and Anesthesia

In total, 16 New Zealand White rabbits weighing 3-4 kg were studied. All animals were kept in a single room and fed a dried diet and water ad libitum. The sample size chosen was based on our previous similar studies [8]. First 0.8 mL of 0.2% Acepran with the active ingredient of Acepromazine was injected intramuscularly. The animal was then induced into general anesthesia with a 3 mL solution containing a mixture of 2.2 mL Ketamine (União Química Farmacêutica Nacional S.A., Embuguaçú, São Paulo, Brazil) and 0.8 mL Xylazine. This was split into 2 equal doses of 1.5 mL intramuscular injections, administered with 3-minute intervals.

### 2.4. Surgical Procedure

**Figure 1:**
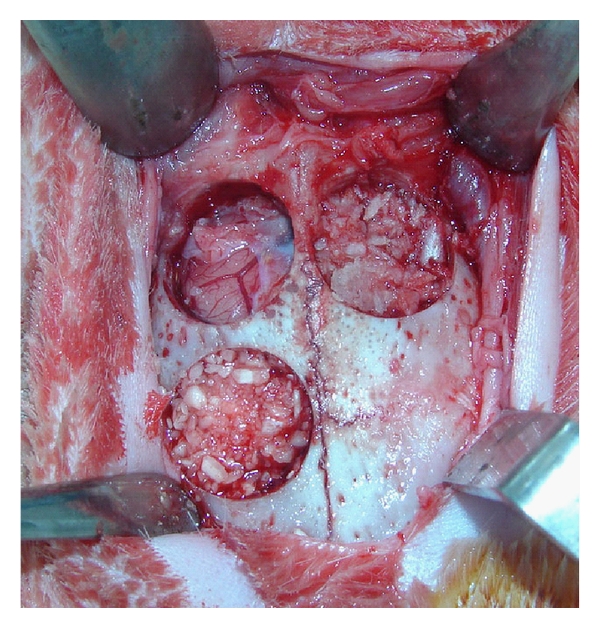
Surgical defects on rabbit calvarium filled with test materials. One defect was left unfilled (upper right), one filled with collected particulate autogenous bone (upper left), and the third defect was filled with the new bovine dentin bone substitute (lower right).

The surgical field was disinfected with povidone-iodine 10%. A mid-sagittal incision was made after local infiltration of 2% lidocaine hydrochloride with 1 : 100,000 epinephrine 1.8 mL. Subperiosteal dissection was carried out, and the periosteum was reflected to expose the bony area. An 8 mm trephine was then used to create 3 circular defects as shown in Figure 1, and the bony discs were removed. It has been shown that 8 mm rabbit calvaria defects can be considered as critical size defects [9].

The defects were chosen randomly, and one defect was filled with the dentin substitute, one was filled with particulate autogenous bone, which was made from crushing the elevated bony discs using a bone mill, and finally the third defect had no filler placed and was left to heal with blood clot only. The defects were filled so as to be in consistent contour with the surrounding bone (Figure 1). No membrane was used in this study. The surgical site was then closed using a 5–0 Nylon suture.

Following the surgery, the animals were administered post-op analgesics (Tramal 0.02 mg/kg, Biolab Searle, São Paulo, Brazil) and anti-inflammatory medication (Profenids 3 mg/kg, Distribuidora Farmácia Brasil LTDA, Jandira, SP, Brazil) intramuscularly.

### 2.5. CT Scan

A multislice CT scan (Siemens Emotion) was then taken immediately postoperatively, and a multislice CT scan was taken immediately after sacrifice, using the same CT scanner.

### 2.6. Sacrificing Procedure

7 rabbits were sacrificed 1 week postoperatively, and 9 rabbits were sacrificed 6 weeks postoperatively. The animals were killed using an overdose of xylazine and ketamine, and the tissues harvested from the graft sites were processed for histology.

### 2.7. Histology

The tissues were fixed by means of perfusion with paraformaldehyde 4% using a peristaltic pump (Masterflex Pump Controller, Cole-Parmer, USA). The biopsies were kept in 4.0% paraformaldehyde solution at 4°C for six hours in order to complete the fixation, then washed in phosphate buffer solution 0.1 M for 30 minutes, and moved on to EDTA 5.0% for decalcification. The pieces were rinsed in phosphate buffer for 24 hours and subsequently kept in solution containing sucrose 30% for two more days. After cryoprotection was concluded, each piece was fixed in a gel support (Tissue TeK—Laboratório FK Biotec, Bento Gonçalves, Brazil), frozen at −20°C, and sliced in a cryostate (Microm HM 505 N), 14 *μ*m thick. The slices were made up in a semiserial fashion in a coronal orientation and assembled on polarized blades, where they were interspersed for the histological analysis. The histological slides were stained with Mallory's trichrome and examined under a light microscope. The histological appearance was assessed by more than one investigator blinded to the nature of the material the defect had been filled with. Where there were differences in the histological assessment of the tissues between investigators, these were resolved by discussion and a consensus reached before blinding was removed.

## 3. Results

The skin wounds healed well with no sign of infection or adverse reaction to the implanted material.

### 3.1. CT Scan

**Figure 2:**
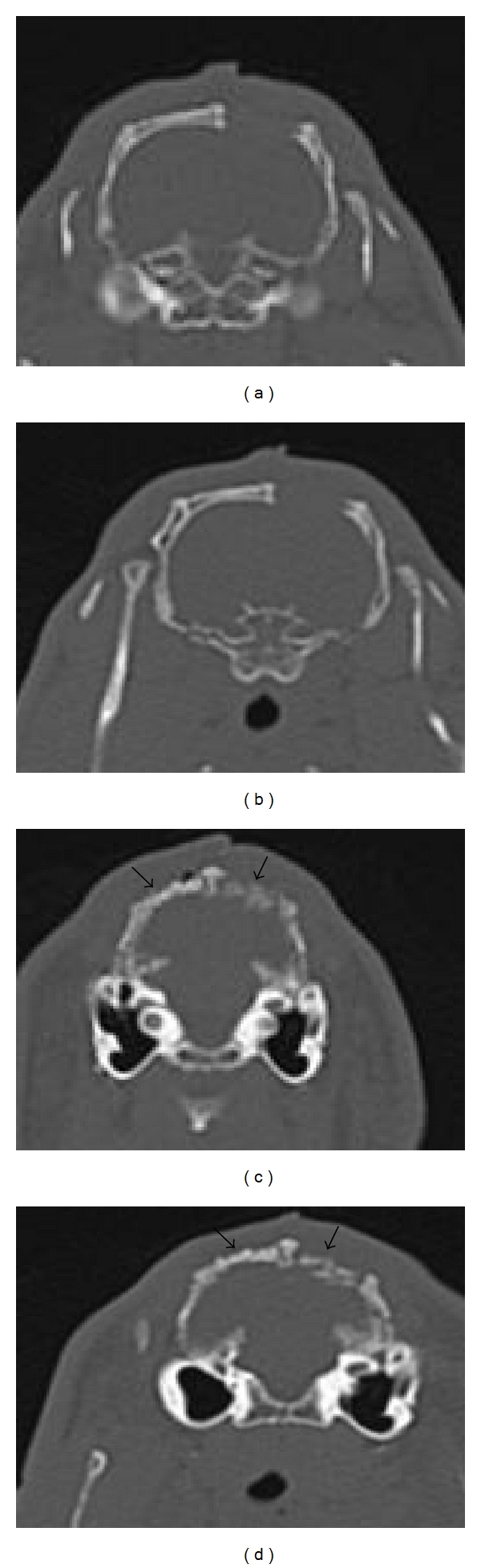
Post-op CT scan of (a) unfilled area immediately after surgery; (b) unfilled area 1 week post operatively; (c) dentin-filled defect (left arrow) and the autogenous bone-filled defect (right arrow) immediately after surgery; (d) dentin-filled defect (left arrow) and the autogenous bone-filled defect (right arrow) 1 week postoperatively.

The immediate postoperative and 1-week postoperative CT scans of defects are shown in Figure 2. After one week, there was no bony reunion or growth in the empty defect. In the filled defects, the dentin had a higher mineral content showing a higher density than the autologous bone when viewed on the CT scan both at the time of surgery and after 1 week.

**Figure 3:**
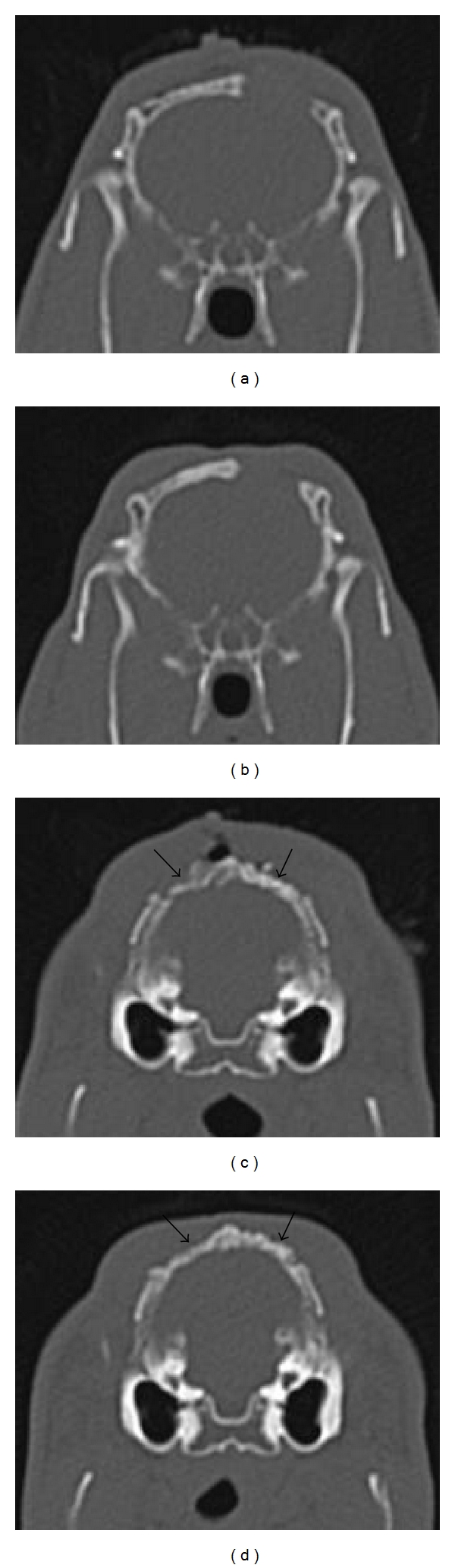
Post-op CT scan of (a) unfilled area immediately after surgery; (b) unfilled area 6 weeks postoperatively; (c) dentin-filled defect (right arrow) and the autogenous bone-filled defect (left arrow) immediately after surgery; (d) dentin-filled defect (right arrow) and the autogenous bone-filled defect (left arrow) 6 weeks postoperatively.

The immediate postoperative and 6-week postoperative CT scans of defects are shown in Figure 3. After 6 weeks, it seemed the empty defect had some appositional bone growth but without any bony reunion. The filled defects both appeared to undergo organisation, with the dentin-filled defect being shown to be more opaque than the autologous graft after 6 weeks.

### 3.2. Histology

**Figure 4:**
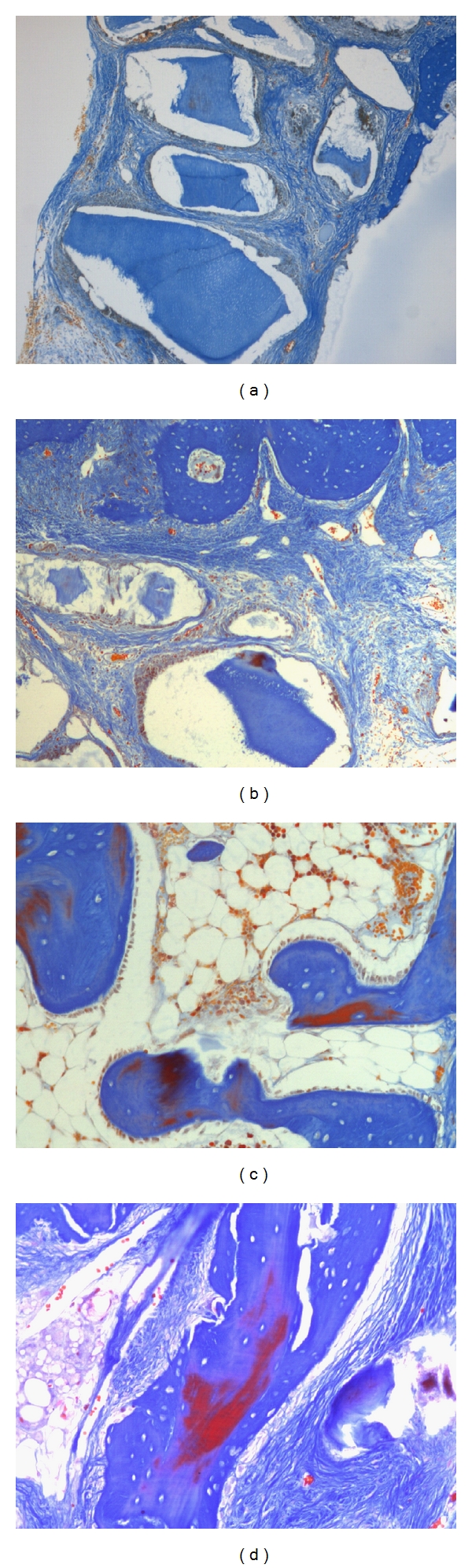
Mallory's trichrome-stained histological sections of defect areas filled with (a) processed dentin product, 1 week post-op, original magnification ×10; (b) processed dentin product, 6 weeks post-op, original magnification ×10; (c) autogenous bone, 1 week post-op original magnification ×20; (d) autogenous bone, 6 weeks post-op, original magnification ×20.

Histological views of the calvarial defects filled with the dentin product and autologous bone after 1 and 6 weeks are shown in Figure 4.

Histological examination of the defects filled with the dentin product revealed the presence of dentin particles after 1 week and 6 weeks. In some areas, the dentin had been partially resorbed showing features of fibrous tissue encapsulation.

Examination of the defects filled with autologous bone showed the presence of osteoblasts on the margin of the bone particles with the evidence of new bone formation. Some soft tissue invasion into between the bone particles was observed.

The untreated defects did not show any significant bony infill after 1 week or 6 weeks and were mainly filled with the soft tissue (figure not shown).

## 4. Discussion

Advances in tissue engineering and stem cell science have led to development of novel approaches for bone regeneration in maxillofacial region [10–12].

Dentin has been of an area of interest to study its potential use as a bone substitute since it has a higher mineral content than any bone-derived material, and it is also a readily available xenograft. Furthermore, there is a potential to utilize its organic component as well as its mineral component. For a long time, it has been known that proteins of similar weight to bone morphogenic proteins (BMPs) are abundant in tooth substance [13, 14] and that the BMPs are able to promote the differentiation of mesenchymal cells into odontoblasts and ameloblasts [15, 16]. Also dentin has the capability of promoting heterotopic bone formation [17]. These proteins may enhance the osteoinductive properties of the bone substitute if they are able to be retained during the processing of the material studied in this experiment. With the prospect of the possible use of bovine dentin as a graft material comes the opportunity to utilize a patient's own dentin from their extracted teeth, which is analogous to a previous study done using the extracted teeth from a rabbit to repair a calvarial defect made in the same animal once the dentin was processed [18]. The clinical scenarios for taking advantage of such an opportunity prior to implant placement are numerous.

Among different models, the rabbit calvarial model has been used for many years as a reliable method of evaluating bone substitutes. This model has been reported to be very suitable for the assessment of osteoconductive properties of biomaterials [19].

The rabbit model has several advantages, such as standardization of experimental conditions and experiment repeatability, size, inexpensiveness, and high bone turnover rate [20]. The calvaria and the facial bones are pure membranous bones, with the mandible and the greater wing of the sphenoid being exceptions. Subtle differences exist between the microscopic structures and functions of the calvaria in different species; however, embryonic development is very similar [7].

The skull is much more biologically inert due to its poor blood supply and relative deficiency of bone marrow, when compared to other bones [21]. There is no primary nutrient artery in the human calvaria, unlike many long bones that contain a primary nutrient artery. Since a large area of human skull is devoid of muscle insertions, the blood supply to the human calvaria is poorer than in other mammals [7]. The resultant effect means that even small defects in the adult human skull do not spontaneously repair. Due to this, the regenerative capacity of the calvaria of experimental animals can be considered better than that of humans. As previously mentioned, calvarial wound defect model has many similarities to the maxillofacial region. Physiologically, the cortical bone in the calvaria resembles an atrophic mandible [22]. Implantation into a skull defect is the most severe test for a bone implant [7], and thus the calvaria has been a frequent site for the testing of bone repair materials. This is unlike the rat femur, for example, which has mainly cancellous bone surrounding the 5-wall defect in the implantation test model.

There are no previously published studies that have used CT data to assess dentin when used as a bone substitute. Multislice computerised tomography data was used as a visual aid to determine the density and the form of the grafted area as it is a readily available noninvasive technique with high accuracy [23]. Furthermore, with newly available software, it is possible to reconstruct a 3D high-resolution image using the data from the scanned subject for easy visual, quantitive, and density analysis. Using the same CT scanner for all the scans, which was calibrated prior to scanning, it was possible to ensure the consistency in the recording of data in the subjects and obtain a realistic comparison of the dentin substitute to the neighbouring empty defect and the defects grafted with autologous bone. This was an ideal way of visualizing the differences in the graft density in this study.

Histological data has the benefit of allowing us to observe a number of features depending on the type of stain that is used. In this study, Mallory's trichrome staining was used. This was a suitable stain for identifying the newly formed bone following decalcification of the specimens. The histological features observed in this study were different from our previous findings using a rat femur implantation testing where complete integration of the bovine dentin to the bone was observed [5]. However, in this study, we unexpectedly found soft tissue encapsulation of the dentin particles. The dentin particles also appeared to be undergoing resorption within the fibrous tissue.

In the previous study using the rat femur model (5-wall defect), there was plenty of cancellous bone in the 5 bony walls, which had a higher blood supply and more osteoblastic cells to repair and deposit new bone around the particles. There was also less soft tissue contact with the graft material, and so, in a 5-wall defect, the likelihood of soft tissue invasion was very low. However, in a large rabbit calvarial defect (4-wall), most of the material was exposed to the soft tissue below and above and was only exposed to bone on the edge of the wound. Also, there was less cancellous bone in a calvarial defect compared to rat femur.

A crestal incision was used to access the surgical site. This meant that the closure site was very close to the defects and material. In hindsight, a remote incision would have been more favorable to minimize the potential risk of skin cells invading the defects. Also, there may have been potential damage to the underlying dura which could account for the soft tissue invasion and encapsulation of the dentin particles.

Whilst this study may have given a negative result as for the suitability of bovine dentin for use in a 4-wall defect, there may still be the potential for its use in smaller 5-wall defects.

The results of histology also showed that there was evidence of some soft tissue invasion into defects filled with autologous bone. Previous studies [18] have shown that, when bone materials are used in a rabbit calvarial model with the presence of a membrane, the bone regeneration appeared more extensive and more matured histologically than bone materials without a membrane. Therefore, future studies in this area may be necessary to explore the means to reduce the soft tissue invasion and promote bone regeneration. This may include the modification of the existing dentin product and the use of guided tissue regeneration approaches.

## 5. Conclusions

The rabbit calvarial defect model used in this study is a suitable and robust model for the assessment of osteoconductive and osteogenic properties of bone materials and indicates that the processed bovine dentin product may not be an ideal bone material for large 4-wall defects. However, based on our previous rat femur implantation studies, the bovine dentin material may be suitable for the repair of smaller 5-wall defects. The findings of this study also confirm that the soft tissue invasion can also occur in large 4-wall rabbit calvarial defects filled with autologous bone.
